# Empirical evaluations of analytical issues arising from predicting HLA alleles using multiple SNPs

**DOI:** 10.1186/1471-2156-12-39

**Published:** 2011-04-25

**Authors:** Xinyi Cindy Zhang, Shuying Sue Li, Hongwei Wang, John A Hansen, Lue Ping Zhao

**Affiliations:** 1 Division of Public Health Sciences, Fred Hutchinson Cancer Research Center, 1100 Fairview Ave N, Seattle, WA 98109, USA; 2 Division of Clinical Research, Fred Hutchinson Cancer Research Center, 1100 Fairview Ave N, Seattle, WA 98109, USA; 3 School of Medicine, University of Washington, 1959 NE Pacific Street, Seattle, WA 98195, USA

## Abstract

**Background:**

Numerous immune-mediated diseases have been associated with the class I and II HLA genes located within the major histocompatibility complex (MHC) consisting of highly polymorphic alleles encoded by the HLA-A, -B, -C, -DRB1, -DQB1 and -DPB1 loci. Genotyping for HLA alleles is complex and relatively expensive. Recent studies have demonstrated the feasibility of predicting HLA alleles, using MHC SNPs inside and outside of HLA that are typically included in SNP arrays and are commonly available in genome-wide association studies (GWAS). We have recently described a novel method that is complementary to the previous methods, for accurately predicting HLA alleles using unphased flanking SNPs genotypes. In this manuscript, we address several practical issues relevant to the application of this methodology.

**Results:**

Applying this new methodology to three large independent study cohorts, we have evaluated the performance of the predictive models in ethnically diverse populations. Specifically, we have found that utilizing imputed in addition to genotyped SNPs generally yields comparable if not better performance in prediction accuracies. Our evaluation also supports the idea that predictive models trained on one population are transferable to other populations of the same ethnicity. Further, when the training set includes multi-ethnic populations, the resulting models are reliable and perform well for the same subpopulations across all HLA genes. In contrast, the predictive models built from single ethnic populations have superior performance within the same ethnic population, but are not likely to perform well in other ethnic populations.

**Conclusions:**

The empirical explorations reported here provide further evidence in support of the application of this approach for predicting HLA alleles with GWAS-derived SNP data. Utilizing all available samples, we have built "state of the art" predictive models for HLA-A, -B, -C, -DRB1, -DQB1 and -DPB1. The HLA allele predictive models, along with the program used to carry out the prediction, are available on our website.

## Background

The genes encoding the human leukocyte antigens, HLA-A, -B, -C, -DRB1, -DQB1 and -DPB1, located within the major histocompatibility complex (MHC), play critical roles in immunity and host defense, risk of autoimmune disease and cancer [1-4]. HLA also plays an important role in organ and cellular transplantation where mismatched HLA can lead to graft rejection and graft-versus-host disease [5,6]. HLA genes are among the most polymorphic systems in the human genome.

Due to the complex and redundant nature of the sequence variations that distinguish class I (HLA-A, -B, -C) and class II (HLA-DRB1, -DQB1 and -DPB1) alleles, which are located within 2 to 3 hypervariable regions in the class I (exon 2 and 3) and class II (exon 2) genes, and the ambiguities inherent in analyzing unphased sequences from heterozygous individuals, high resolution HLA genotyping remains labor intensive and costly. Recently, developments in SNP genotyping have enabled many large-scale genome-wide association studies (GWAS) that have generated genotype data for thousands of SNPs in the MHC region. It has been demonstrated that the MHC SNPS can be used to predict HLA alleles [7,8]. Building upon the success of these early works, we have developed a complementary method that builds predictive models for HLA alleles based on available unphased MHC SNP datasets obtained from unrelated individuals whose HLA alleles are known [9]. The methodology uses a likelihood framework of all HLA and SNP genotypes in the sample, systematically identifies the most informative SNPs, and has succeeded in incorporating predictive models capable of a very high rate of accuracy sufficient for deducing HLA alleles in disease association GWAS datasets.

To optimize the practical value of these HLA predictive models, one needs to address the following questions: (1) can imputation of untyped MHC SNPs improve the accuracy and robustness of the model; (2) can MHC SNPs generated by different platforms yield comparable HLA allele predictions; (3) are prediction models transferable across different study populations; and (4) should multi-ethnic groups be included in the training set when building predictive models? In this manuscript, we explore these questions and make practical recommendations based on empirical data. Finally, we have integrated all available data resources and produced a set of models for predicting HLA-A, -B, -C, -DRB1, -DQB1 and -DPB1alleles.

## Methods

### Study populations

We utilized readily available SNP genotype data from a cohort of normal donors who participated in a GWAS at the Fred Hutchinson Cancer Research Center (FHCRC), control samples from the British 1958 Birth cohort in Welcome Trust Case Control Consortium (WTCCC), and participants in Phase IIb clinical trial on HIV1-vaccine (STEP). Characteristics of these cohorts are summarized in Table 1.

**Table 1:** Characteristics of the three study cohorts

		FHCRC	WTCCC*	STEP
	Caucasian	1280	2730	411
	Mestizo/Mestiza			212
Ethnicity	Others (African, Asian and other ethnic groups)	226		209

	HLA-A, -B, and -C	1483	1929,1812,1558	832
HLA Loci	HLA-DRB1	1481	1969	832
	HLA-DQB1	1434	1965	
	HLA-DPB1			832

	Affy 5.0 array	1506		
SNP genotyping platforms	Affy 500K		1480	
	Affy 6.0 array		2706	
	Illumina 550K		1438	
	Illumina 1.2M		2692	832

* In the WTCCC cohort, dubious samples were excluded according to WTCCC suggestions.

Number of samples in each study cohort that were of each ethnicity, genotyped for each HLA loci and genotyped on each SNP genotyping platform.

#### FHCRC

This cohort consists of ~1,500 normal donors who were genotyped on the Affymetrix 5.0 Human SNP Array, and also genotyped for HLA-A, -B, -C, -DRB1 and -DQB1 alleles. A systematic quality control (QC) has been performed: to ensure genotyping quality, the study genotyped 152 duplicate samples, allowing us to assess genotype concordances. Based upon our duplicate samples, the Affymetrix-defined QC call rate threshold was raised from 86% to 90%, to ensure that the genome-wide genotype concordance exceeded 99%. Further, we have ruled out any unintended sample duplication by computing identity-by-state between all pairs of samples. Furthermore, we have also inferred gender using SNPs, and computed their concordance with the recorded gender. Finally, a series of usual QC matrices (missing data percentages, minor allelic frequencies and departure from Hardy-Weinberg equilibrium) are computed and used to filter undesirable SNPs.

#### WTCCC

As part of the WTCCC study, ~3,000 controls from the British 1958 Birth cohort were genotyped on both the Affymetrix 6.0 and Illumina 1.2M chips. About half of these samples were also genotyped on the Affymetrix 500K chip and Illumina 550K chip in WTCCC1 studies [10]. All samples are of Caucasian descent, and all were genotyped for HLA-A, -B, -C, -DRB1 and -DQB1 alleles.

#### STEP

A Phase IIb HIV-1 vaccine clinical trial referred to as the Step Study consisting of ~3,000 samples from several different countries [11]. Among participants, 832 participants were genotyped on Illumina 1.2M, and were genotyped for HLA-A, -B, -C, -DRB1 and -DPB1 alleles. A notable feature of this group is that approximately 25% of participants are Mestizos of European and American Indian ancestry. Following the same QC protocol described above, we computed the usual QC metrics, and filtered out undesirable SNPs accordingly.

In addition to the SNP genotyping quality control originally performed by each study group, we censored by filtering SNPs with minor allele frequency less than 0.01, SNPs missing in more than 5% of the samples, and SNPs which deviated from Hardy-Weinberg equilibrium with p value less than 1e^-20^. All HLA alleles were genotyped using high quality sequencing methods as discussed below.

### A Brief Introduction to the HLA Prediction Methodology

A general methodology of building predictive models for polymorphic genes using SNPs has been detailed elsewhere [9]. Briefly, this methodology includes a training procedure to produce one predictive model for each HLA locus, and a separate procedure to validate the predictive model. During the training process, a set of SNPs are selected to best predict HLA alleles. The selection is carried out by using the forward-and-backward scheme, which starts with the SNPs within a HLA gene (if available) and gradually extends to flanking regions of each HLA gene. The search region is empirically chosen to maximize the prediction accuracies while retaining parsimony. The selection process is evaluated by an objective function, which is the negative log-likelihood of the HLA allele given SNP genotypes penalized by the number of haplotype parameters to be estimated.

The predictive model is validated with an independent set of samples whose MHC SNPs and HLA alleles are known. Given the SNP genotypes, probabilities of all possible pairs of HLA alleles are assigned according to the predictive model. The pair with the maximum probability is predicted to be the HLA allele for that sample, if the probability exceeds a pre-specified confidence threshold (CT). Typically, CT is set at 0, which means that all samples will be predicted, i.e. the call rate is 100%. If CT is chosen to be any value greater than 0, such as CT = 0.5 or 0.9, only the samples with the maximum predictive probability exceeding CT will be assigned an HLA allele specificity. In this manuscript, we will only list the results of call rate if CT > 0. The accuracy of prediction is estimated by comparing predicted HLA alleles with the known HLA genotype.

The training set consisted of 630 healthy unrelated individuals in the FHCRC cohort and the predictive models were validated with a separate set of 630 healthy unrelated individuals in the FHCRC cohort. The predictive models achieved accuracies for HLA alleles defined at intermediate resolution (2 digit) or high resolution (4 digit) ranging as high as 97% and 95% for HLA-A, 96% and 93% for HLA-B, 98% and 97% for HLA-C, 93% and 79% for HLA-DRB1 and 97% and 83% for HLA-DQB1 with CT = 0.

### HLA Allelic Calls in Three Cohorts

For the FHCRC cohort, HLA genotyping was initially performed using sequence specific oligonucleotide probes (Dynal RELITM SSO - HLA Class I & II Strips Line strips; and the Luminex-based by One Lambda LABType^® ^SSO) followed by sequencing of exon 2 and 3 for the class I HLA-A, B and C genes, and sequencing of exon 2 for the class II HLA-DRB1 and DQB1 genes. The same sequencing technology was used to genotype HLA-A, -B, -C, -DRB1 and -DPB1 for the STEP. HLA-A, -B, -C, -DRB1 and -DQB1 genotyping for the WTCCC cohort was done using SSOP reagents from Invitrogen. WTCCC investigators have resolved ambiguous allele calls to the level of a series of alternate alleles (Additional file 1: Table S1a) as explained on their website (https://www-gene.cimr.cam.ac.uk/todd/public_data/HLA/HLA.shtml).

Having demonstrated the high prediction accuracy of our HLA allele prediction models in [9], we addressed relevant practical issues for optimizing this methodology. In order to answer the questions raised in the Background Section, the HLA nomenclature used for the three cohorts has to be adjusted to compensate for the differences in the coding of ambiguous allele calls across the three cohorts without distorting HLA allele integrity (Additional file 1: Table S1b). As expected, these ambiguous sets of HLA alleles represent a source of reduced prediction accuracies, regardless of predictive methodologies. Samples with missing HLA genotypes or unresolved ambiguities were excluded from the analysis.

### Imputation

We used IMPUTE v2 (https://mathgen.stats.ox.ac.uk/impute/impute.html) to impute missing SNPs from the HapMap project. Default parameters were applied to impute only SNPs in the extended MHC region of chromosome 6 (28,799,220-34,204,868) [12]. Our study samples included individuals from several ethnically distinct populations that are not represented in the HapMap panel. For these minority populations, combining the reference groups in the HapMap is usually preferred as previously demonstrated [13,14]. Li et. al. [13] also showed that even when the ethnicity-specific panel is preferred, the error rate for using the combined reference panel increases by only 0.15%. Therefore, for data consistency and optimal imputation performance in all ethnic groups, we chose to use genotype data from HapMap release 22 with three ethnic groups (Caucasian, African and Asian) as the imputation reference panel. For each imputed SNP, the genotype was called if its posterior probability exceeds 0.8. The imputed SNPs were then further filtered if their call rate is less than 95% or if the minor allele frequency is <1%.

### Usefulness of Imputed SNPs

SNP imputation has become a routine practice in genome-wide association studies. Frequently used as the imputation reference panel, the HapMap data (http://hapmap.org) includes ~2.4 million SNPs on the whole human genome and 10,600 SNPs in the MHC region, where typical commercial genotyping arrays have only ~2,000 SNPs. Increasing the number of SNPs used in predicting HLA alleles may increase accuracy. In addition, a common set of HapMap SNPs, independent of genotyping array technologies, would allow us to build general predictive models independent of genotyping technologies. However, this option has potential limitations. First, the use of imputed SNPs may not provide independent information, since SNPs are imputed based on the local linkage-disequilibrium (LD) information, which has already been incorporated via tagging SNPs selected on commercial arrays. Further, prediction accuracies of less common haplotypes may be negatively impacted by restricting haplotype polymorphisms to those within the HapMap samples.

Hence, to evaluate the utility of imputed SNPs in building predictive models, we have empirically investigated its pros and cons. In this empirical evaluation, we used SNP genotype data from 1,260 Caucasian samples from the FHCRC cohort which were genotyped on the Affy 5.0 array, and used half of the samples as the training set and the other half as the validation set. To retain the data independence, we performed imputations for the training set and the validation set separately. For each of the HLA-A, -B, -C, -DRB1 and -DQB1 loci, we trained and validated the HLA predictive model using the set of observed SNPs only, and then using the set with both observed and imputed HapMap SNPs. To compare the two sets of models trained with or without imputed SNPs, we introduce a summary statistics as the average difference in prediction accuracies among the five HLA loci between these two sets of models. The performance of prediction is evaluated at both intermediate and high resolution using CT = 0, 0.5, and 0.9.

Recognizing that the Affy 5.0 array having the least number of SNPs in the MHC region (Table 2) could be a special case, we repeated the above experiment protocol to assess the usefulness of imputed SNPs when SNPs were genotyped on Affy 500K, Affy 6.0 array, Illumina 550K, and Illumina 1.2M, using the WTCCC control data. In this exercise, we used 1,001 samples that were genotyped on all four technologies, per WTCCC suggestion, and that had complete genotypes on HLA-A, -B, and -C alleles. Randomly dividing 1,001 samples into training and validation sets, and using genotypes from each array technology, we repeated the same training and validation calculations using observed SNPs only, as well as using both observed and imputed HapMap SNPs.

**Table 2:** Numbers of overlapping SNPs between HapMap project and four genotyping platforms in the extended MHC region ^1^

	HapMap	Affy 500K	Affy 6.0	Illumina 550K	Illumina 1.2M
HapMap	10600	1307	1979	1762	4382
Affy 500K		1422	1363	272	610
Affy 6.0			2325	459	988
Illumina 550K				1944	1885
Illumina 1.2M					6303

^1 ^The range of extended MHC region is chr6: 28,799,220 - 34,204,868 [12].

### Different Technologies

Genome-wide DNA array genotyping technologies are rapidly converging to the Affymetrix and Illumina technologies, each of which has gone through several different array formats, e.g., Affy 500K and 6.0, and Illumina 550K and 1.2M. Different sets of SNPs are placed on these arrays, and the overlap between each pair of technological platforms is quite small (Table 2). To facilitate the comparison of HLA predictive models built using SNPs of different technologies, we performed imputation on the datasets generated by each technology to obtain genotypes of the common set of SNPs used in the HapMap project. With the common set of SNPs, we can address two questions: are genotypes comparable between technologies; and are prediction accuracies comparable between the predictive models built using the genotypes from different technologies?

To address these questions, we used the same set of 1,001 Caucasian samples from the WTCCC cohort which were genotyped on four different technologies. These samples were divided into training and validation sets. To assess the concordance of genotype data between technologies, we computed the Cohen's kappa coefficient, a commonly used index to quantify agreement between two measurements [15]. It takes into account the concordance by chance and is calculated as *κ *= [Pr(*a*) -Pr(*e*)]/[1-Pr(*e*)], where Pr(*a*) is the observed agreement percentage, and Pr(*e*) is the chance agreement percentage. The larger the kappa coefficient, the better the concordance is between two measurements, with *κ *approaching 1 as the perfect agreement. Otherwise, the kappa coefficient approaches zero. For each pair of technologies, we assessed the concordance between all genotypes of SNPs that are observed in both, SNPs that are observed in one but imputed in the other, SNPs that are imputed in both, and the overall HapMap SNPs. Any SNPs which were missing on one of the paired platforms were excluded.

To address the second question, we built predictive models on the training set using the HapMap SNPs observed or imputed from each genotyping technology. Then, we compared their accuracies of prediction on the validation set using SNPs from each of the four genotyping technologies at both intermediate and high resolution for each HLA locus.

### Across Populations

Besides the technological heterogeneity, another issue is whether the predictive models are transferable across different study populations of the same ethnic group, say, the FHCRC and WTCCC populations of Caucasian descent. To address this question, we built predictive models using both imputed and observed HapMap SNPs of all Caucasian samples in the FHCRC cohort, and tested these predictive models in the WTCCC cohort. Vice versa, we also tested WTCCC predictive models on the Caucasian samples of the FHCRC cohort. To minimize the possible impact of different SNP genotyping platforms, we used the subset of samples in the WTCCC cohort that were genotyped on the Affy 500K array, which is comparable to the Affy 5.0 array used in the FHCRC cohort.

### Building Models Using Multi-Ethnic Samples

HLA alleles and their allelic distributions are known to differ substantially between ethnic groups, reflecting their recent evolutionary histories. One natural question is if one should develop ethnic-specific predictive models as opposed to combining all available ethnic groups together to develop "global predictive models". To address this question, we used the samples from STEP, which includes 411 Caucasians, 212 Mestizos, and 209 samples of other ethnicities (including 82 African, 76 Hispanic, and 51 others). We combined three sets of 150 randomly selected samples from each ethnic group to form a training set, and built HLA predictive models for each of the HLA-A, -B, -C, -DRB1, and -DPB1 alleles. Similarly, we also built predictive models using only the 411 Caucasian samples. Both sets of predictive models were validated on the samples genotyped on Illumina 1.2M in the WTCCC study, and the remaining samples from STEP that were not included in the training set.

## Results

### Imputed SNPs

To evaluate the usefulness of imputed SNPs in constructing HLA predictive models, we first used the SNP data from the FHCRC cohort that was generated by the Affy 5.0 array. Table 3 shows accuracy estimates obtained from the validation analysis of predictive models built with and without imputed SNPs. For the predictive models built without including imputed SNPs, the accuracies are consistent to those reported earlier [9], and are slightly higher for high resolution HLA-DRB1 and -DQB1 due to the consolidation of high resolution alleles of the three cohorts. Of these models, the accuracy is highest at HLA-C loci, where 98% of the HLA alleles at intermediate resolution, and 97% at low resolution are predicted correctly with CT = 0. By applying a confidence threshold (CT) of 0.9 to the posterior probability of HLA alleles, the highest accuracy is increased to 99% at HLA-A loci at intermediate resolution with call rate 87%. When comparing accuracies between predictive models built with and without imputed SNPs, one observes that accuracies of models with imputed SNPs are the same or higher than those without imputed SNPs, with one exception: accuracy for HLA-DQB1 at intermediate resolution with CT = 0.9 equals 97% with imputed SNPs (call rate 94%), versus 98% without imputed SNPs (call rate 95%). On average, using imputed SNPs improves the prediction accuracy (call rate) by 0.8%, 0.6%(0.4%), and 0.2%(0.6%) at intermediate resolution and 1.6%, 1%(2%), and 0.6%(1%) at high resolution with CT = 0, 0.5 and 0.9 respectively. In general, this empirical result supports the hypothesis that predictive models built with imputed SNPs would perform equally well, if not better, in comparison with models built without imputed SNPs.

**Table 3:** Comparison of prediction accuracies for HLA predictive models built with and without imputed SNPs

		Without Imputed SNPs			With Imputed SNPs		
	HLA-	CT = 0	CT = 0.5	CT = 0.9	CT = 0	CT = 0.5	CT = 0.9
Intermediate Resolution	A	97	97(100)*	99(87)	98	98(100)	99(89)
	B	95	95(99)	98(86)	96	96(100)	98(88)
	C	98	98(100)	98(97)	99	99(99)	99(97)
	DRB1	93	93(99)	97(78)	93	93(100)	98(78)
	DQB1	96	97(99)	98(95)	97	97(100)	97(94)

High Resolution	A	95	95(100)	97(86)	97	97(100)	98(86)
	B	93	93(98)	96(78)	93	94(97)	96(72)
	C	97	97(99)	98(95)	98	98(100)	98(93)
	DRB1	83	87(85)	94(48)	87	88(95)	95(59)
	DQB1	94	95(100)	95(94)	95	95(100)	96(96)

* Prediction accuracy % (call rate %)

Using the Caucasian samples genotyped on the Affy 5.0 array in the FHCRC cohort, the accuracies of the predictive models built with the training set (N = 633) were

evaluated on the validation set (N = 627) for HLA-A, -B, -C, -DRB1 and -DQB1 at intermediate and high resolution, with CT = 0, 0.5 and 0.9.

**Figure 1:**
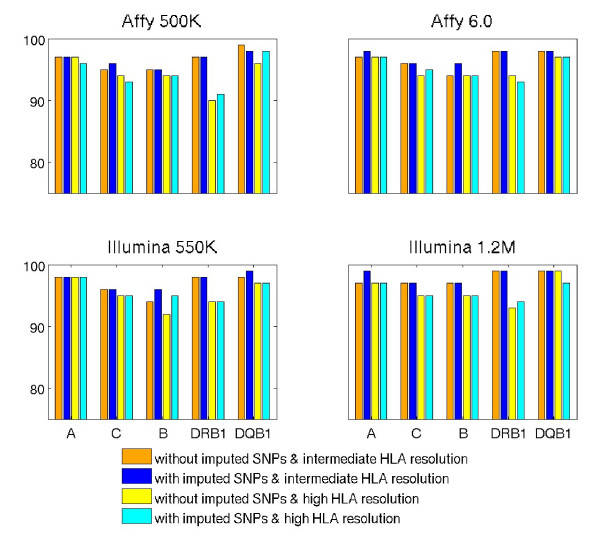
**Comparison of prediction accuracies between models built with and without imputed SNPs from four arrays**. Half of the common set of samples genotyped on Affy 500K, Affy 6.0, Illumina 550K, Illumina 1.2M arrays in the WTCCC cohort were used as the training set (N = 501) and the other half were used as the validation set (N = 500). Each panel shows a comparison of prediction accuracies for the validation set, with models built using only SNPs observed from the array or using HapMap SNPs observed and imputed from the array. The confidence threshold was set at CT = 0.

To extend this observation beyond Affy 5.0, we used the WTCCC data, to evaluate the usefulness of imputed SNPs from Affy 500K, Affy 6.0, Illumina 550K, and Illumina 1.2M. Figure 1 has four panels of figures, labeled by the corresponding technologies. Comparing accuracies between models built with and without imputed SNPs, one would conclude that predictive models built with imputed SNPs again have either comparable or better accuracies, with few exceptions. For those exceptions, losses of accuracies are less than 1%. Certainly, variations between two classes of models are much less than those between intermediate and high resolutions. Details in accuracies of prediction across different CTs are listed in the Additional file 1: Table S3.

### Across Technologies

Realizing that there are multiple genotyping technologies, one cannot help but wonder whether using a particular genotyping technology would lead to the desired HLA allele predictions. To address this question, we first assess if genotype data from different technologies are comparable. Table 4 lists kappa coefficients between paired technologies, with respect to directly measured genotypes and imputed genotypes. Comparing two Affymetrix arrays, one observes that the kappa coefficient is as high as 99.69% for genotypes observed on both arrays, which demonstrates high genotyping quality. The kappa coefficient for I-O (genotypes Imputed on Affy 500K and Observed on Affy 6.0) is 98.60%, which is the lowest among all four comparisons. This level of concordance is nevertheless quite remarkable, because it shows high imputation quality even though nearly 10,000 SNPs are imputed on Affy 500K in this 6 Mb region. Concordance of SNPs imputed on both platforms is nearly 99.78%, partially reflecting the shared reference panel used in the imputations. Similar concordance is observed for two Illumina arrays. Across two commercial platforms, concordance between Affy 6.0 and Illumina 1.2M is high, and equals 99.70%, 99.47%, 99.13% and 99.76% for O-O, O-I, I-O and I-I, respectively. In general, concordance between paired technologies is outstanding and ranges from 99.31% to 99.77%.

**Table 4:** Kappa coefficients of observed or imputed SNP genotypes between genotyping platforms using the WTCCC cohort data

Platform Pair	O-O^*^	**O-I**^**+**^	I-O	I-I	**Overall**^**#**^
Affy 500K & Affy 6.0	0.9969	0.9901	0.9860	0.9978	0.9968
Illumina 550K & Illumina 1.2M	0.9996	0.9945	0.9943	0.9988	0.9977
Affy 6.0 & Illumina 1.2M	0.9970	0.9947	0.9913	0.9976	0.9955
Affy 500K & Illumina 550K	0.9976	0.9942	0.9847	0.9959	0.9942
Affy 500K & Illumina 1.2M	0.9962	0.9956	0.9876	0.9954	0.9931
Illumina 550K & Affy 6.0	0.9979	0.9897	0.9934	0.9978	0.9962

* O stands for observed genotypes of HapMap SNPs;

+ I stands for imputed genotypes using IMPUTE v2 with HapMap reference panel of Caucasian, African and Asian.

# Overall stands for observed/imputed genotypes of HapMap SNPs;

**Figure 2:**
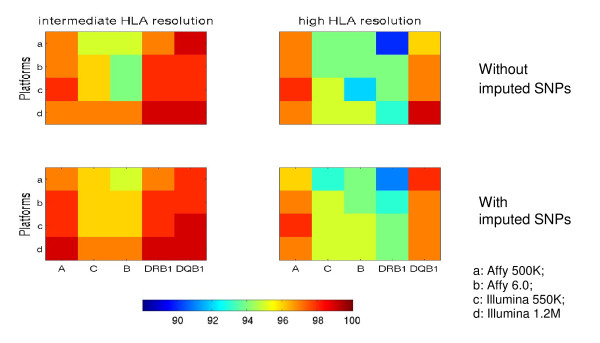
**Comparison of genotyping arrays, with respect to their prediction accuracies**. The training and validation sets were the same as those in Figure 1. Each panel shows a comparison of prediction accuracies of models built with and without imputed SNPs from four genotyping arrays at intermediate and high HLA resolution. The confidence threshold was set at CT = 0.

Now let us examine the prediction performance across technologies. Figure 2 shows accuracies of prediction (in percentage) in the WTCCC cohort with and without imputed SNPs for both intermediate and high HLA resolution predictions with CT = 0. Five HLA loci are listed on x-axis, in the order of their chromosome positions, while four genotyping technologies are listed on the y-axis. As the color bar shows, the red corresponds to higher accuracy, while the dark blue shows lower accuracy. In general, the prediction accuracies are comparable and are above 90%. Among the four genotyping technologies compared, Illumina 1.2M generally performs better for HLA prediction, partly due to having the densest SNPs in the MHC region. However, when building models using only the observed SNPs, Illumina 550K predicts up to 1% more accurately at HLA-A, and high resolution HLA-DRB1 than Illumina 1.2M. By limiting the SNPs from Illumina 1.2M to those also genotyped on Illumina 550K, the prediction accuracies are inferior to the models using all SNPs from Illumina 1.2M (not shown). The slight improvement of Illumina 550K might be attributed to the different SNP contents between the two technologies. For each HLA locus, the variation between different technologies for the two resolution panels with only the observed SNPs is at most 3%. The variation decreases to 2% if imputed SNPs were incorporated in the predictive models. This is not surprising, given that each technology genotyped a different set of SNPs. Using the uniform set of HapMap SNPs minimizes the discrepancy between technologies, and enables one to combine data from different technologies or to use training and validation sets from different technologies.

**Figure 3:**
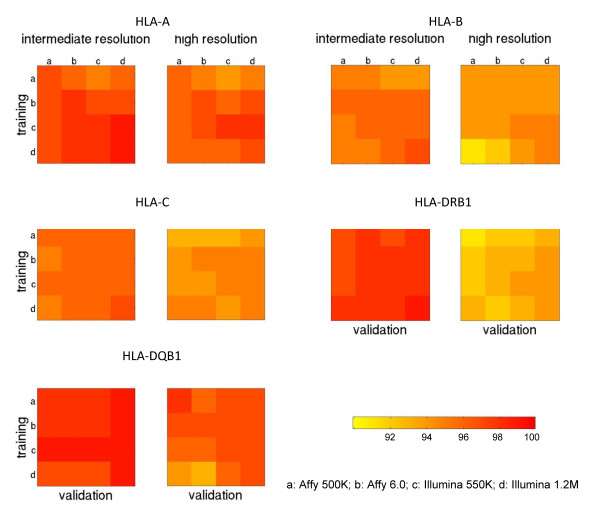
**Comparison of cross-platform prediction accuracies among four genotyping arrays**. Each square panel shows the accuracies of HLA predictive models built using SNPs observed and imputed from one genotyping array and validated using SNPs observed and imputed from another array. The confidence threshold was set at CT = 0.

Results for assessing the performance of predictive models, trained using genotypes observed and imputed from one platform but applied to genotype data observed and imputed from another platform, are shown in Figure 3, which has five panels corresponding to five HLA genes. Within each panel, we show HLA prediction accuracies validated on four technologies (x-axis), when the corresponding model is trained on each of four different data sets (y-axis). The diagonal line shows prediction accuracies when training and validation sets are genotyped on the same technology. As the colored bar shows, red color indicates higher accuracy and yellow color indicates relatively lower accuracy. From visual inspection, when training and validation sets are genotyped on different technologies, predictive models trained using Illumina 550K SNPs seem to slightly outperform models using other platforms for the majority of HLA genes, at both intermediate and high resolutions. However, the accuracies of prediction at each HLA gene are generally comparable among the four genotyping technologies. Detailed accuracy estimates are shown in the Additional file 1: Table S4.

### Across Populations

**Figure 4:**
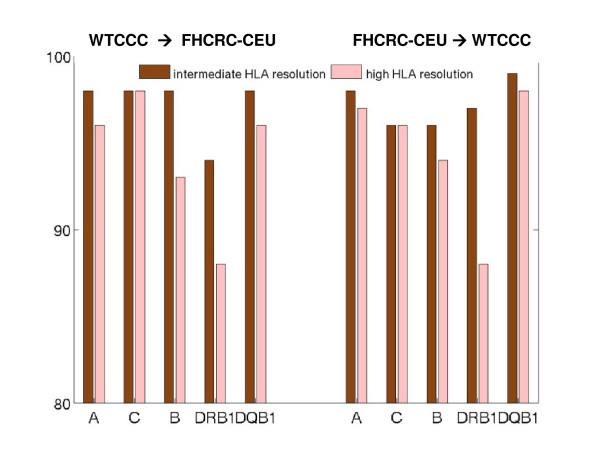
**Comparison of prediction accuracies across populations of the same ethnic group**. The accuracies of HLA predictive models trained on samples from the WTCCC cohort (genotyped on Affy 500K) and validated on Caucasian samples in the FHCRC cohort (genotyped on Affy 5.0), and vice versa. Both observed and imputed HapMap SNPs were applied. The confidence threshold was set at CT = 0.

Predictive models, to be useful, should be reproducible across different study populations of the same ethnicity. To explore this transferability across populations, we built predictive models based on samples in the WTCCC and assessed predictions among Caucasian samples in the FHCRC cohort, referred to as FHCRC-CEU, shown in the left panel of Figure 4. The results of the reversed training and validation populations are shown in the right panel. The call threshold is set at CT = 0. At intermediate resolutions, prediction accuracies for HLA-A, -B, -C, and -DQB1 alleles, from WTCCC to FHCRC-CEU are uniformly around 96%. Even at high resolutions, prediction accuracies are at least 94%. Conversely, from FHCRC-CEU to WTCCC, results are quite comparable. The prediction accuracies for HLA-DRB1 alleles, however, are somewhat disappointing: they are only 88% at high resolution in both prediction exercises. This result may be partially due to the exceptional polymorphism for HLA-DRB1 allele. Another contributing cause could be associated with minor errors in existing HLA databases.

### Use of Multi-Ethnic Samples in Model Building

**Figure 5:**
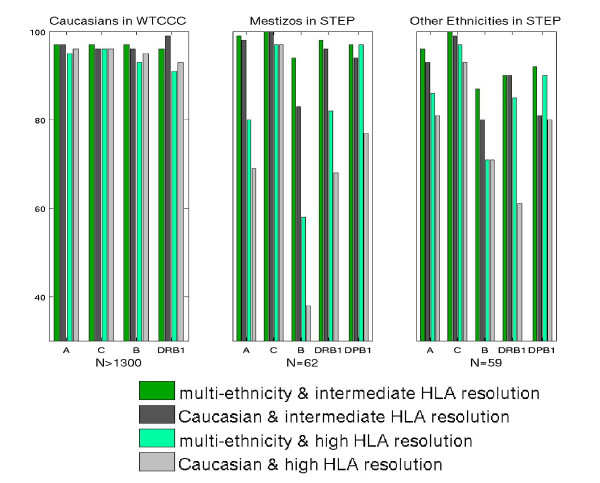
**Comparison of prediction accuracies between ethnic-specific model and multi-ethnic model**. Building predictive models using both observed and imputed HapMap SNPs of multi-ethnicity or Caucasian only samples from the STEP cohort, the prediction accuracies for Caucasian/Mestizo/other ethnicities are shown in each panel. The size of the validation set is shown below each panel. Both the STEP and WTCCC cohort were genotyped on Illumina 1.2M. The confidence threshold was set at CT = 0.

The above exercises have shown that our HLA predictive models are generally transferable among populations of the same ethnicity. However, the majority of current large population studies are on Caucasians, with studies on other ethnic groups being relatively small or limited. Therefore, predictions of HLA alleles in studies of non-Caucasian subjects are forced to use predictive models trained on different ethnic groups, even though predictions are known to be less accurate. An interesting question is whether prediction accuracies will increase if multi-ethnic samples are included in the training set. To assess this question, we utilized the data set from the STEP cohort to train the models on Caucasians only (N = 411) and on multi-ethnic groups (N = 450). Figure 5 shows the validation results with CT = 0 on Caucasian samples genotyped on Illumina 1.2M in WTCCC (left panel), on Mestizo samples who were not included in the training set (middle panel), and on samples of other ethnicities in the STEP cohort but were not included in the training set (right panel). Each panel lists accuracies across all HLA genes (DPB1 is not available from WTCCC). To illustrate the prediction accuracy of each HLA gene, we used green, dark gray, light green and light gray bars, corresponding to the four models resulting from combinations of mixed ethnicity vs Caucasian and intermediate vs high resolution, respectively. For Caucasians in WTCCC (left panel), ethnic-specific models have better accuracies than the multi-ethnic models in general. However the reduction of prediction accuracies of multi-ethnic models is small at 0.25% and 1.25% at intermediate resolution and high resolution respectively with CT = 0.

For Mestizos (middle panel) and other ethnicity groups (right panel), predictive models trained on multi-ethnic samples perform better than those trained on samples of different ethnicity (Caucasian in this case) for all the five HLA loci with CT = 0. For example, for high resolution HLA-A, the accuracy of prediction model trained on Caucasians is only 69%, while improved accuracy of 80% is observed on multi-ethnic populations. For HLA-B, and -DPB1, the improvement is quite dramatic, from 38% to 58% and 77% to 97% respectively. Similar trends have been observed for the mixture of other ethnic groups. If a higher threshold such as CT = 0.5 or 0.9 is applied, the multi-ethnic models still result in greater accuracy, but yield lower call rates, in general. For details, see Additional file 1: Table S5. Of course, results should not be over-interpreted, given the relatively small sample sizes in both training and validation sets (see Discussion).

### Predictive Models for HLA-A, -B, -C, -DRB1, -DQB1 and -DPB1

Based upon empirical explorations of HLA and SNP genotype data from three cohorts, we have come to the following conclusions: 1) use of imputed SNPs is generally beneficial, 2) predictive models are generalizable among genotyping technologies and study populations, and 3) predictive models, built upon multi-ethnic groups, are preferred, especially for samples without ethnic-specific references.

To facilitate applications of HLA predictive models, we combined all available data sets from the FHCRC, WTCCC and STEP cohorts to build general predictive models for HLA-A, -B, -C, -DRB1, -DQB1 and -DPB1. Resulting models along with the program are available on our website http://qge.fhcrc.org/MAGprediction/. On this website, predictive models built using all the Caucasian samples from the FHCRC cohort from our earlier study [9] are also available. Table 5 lists the characteristics of samples in the final training set. From the WTCCC cohort, those genotyped on Illumina 1.2M, about 90% of the entire WTCCC cohort, were included in the training set. Table 6 shows counts and positions of the selected SNPs in the final predictive models, and Additional file 1: Table S6 lists all selected SNPs for each HLA locus at both intermediate and high resolutions. For the remaining 10% of WTCCC samples (283 samples) not included in the training set, we treated them as the validation set, and computed the accuracies of their predicted HLA alleles (Table 6). As shown, accuracies of final predicative models approach 99% at intermediate resolutions and 98% at high resolutions with CT = 0.

**Table 5:** Characteristics of samples in the training set of the final HLA predictive model

Ethnicity	Freq	Array	Freq	HLA-	Intermediate Resolution	High Resolution
Caucasian	4119	Affy 5.0	1483	A	4027	4025
Mestizo	212	Illumina 1.2M	3279	B	3919	3897
Hispanic	137			C	3674	3648
Black	106			DRB1	4063	4032
Other	188			DQB1	3156	3066
				DPB1	832	832

**Table 6:** Selected SNPs in the final HLA predictive models and their prediction accuracies

		Selected SNPs				Accuracy%(Call Rate%)		
			
	HLA-	Count	start	Stop	Length	CT = 0	CT = 0.5	CT = 0.9
Intermediate Resolution	A	33	29,850,894	30,111,284	260,390	98	99(97)	99(89)
	B	57	31,267,324	31,554,345	287,021	95	95(99)	96(90)
	C	36	31,280,634	31,458,359	177,725	99	99(100)	99(93)
	DRB1	33	32,506,503	32,793,404	286,901	98	98(100)	99(81)
	DQB1	20	32,444,139	32,778,222	334,083	98	98(100)	99(94)
	DPB1	64	33,106,707	33,249,258	142,551	-	-	-

High Resolution	A	59	29,918,924	30,171,347	252,423	96	97(95)	97(79)
	B	85	31,370,902	31,561,526	190,624	94	95 (95)	96 (81)
	C	50	31,187,623	31,444,736	257,113	98	98(99)	98(86)
	DRB1	41	32,477,466	32,811,823	334,357	91	94(92)	98(49)
	DQB1	28	32,517,462	32,870,439	352,977	98	98(98)	98(92)
	DPB1	69	33,093,572	33,290,873	197,301	-	-	-

## Discussion

In recent years, GWAS of autoimmune diseases have discovered and validated several SNPs with the MHC region [16,17]. Some of these autoimmune diseases are also known to be associated with HLA alleles. To facilitate the interpretation of MHC-SNP associations in the context of HLA without taking on the costly HLA genotyping, we propose to use HLA predictive models to ascertain HLA alleles using their flanking SNPs. The method we have recently described is capable of achieving a high level of accuracy in predicting HLA-A, -B, -C, -DRB1 and -DQB1 at both intermediate ("antigen") and high ("allele-level") resolutions in Caucasians [9]. In this follow-up study, we have examined several properties of these predictive models of practical importance and have reached the following conclusions.

### Usefulness of Imputed SNPs

Through explorations of the FHCRC and WTCCC data sets, we have found that predictive models built with imputed SNPs usually have greater prediction accuracies, and occasionally have lesser accuracies in a negligible amount. In addition, the use of imputed SNPs enables the potential of general predictive models regardless of genotyping technology, which is of great benefit in terms of increasing sample size and easing the application of predictive models. Therefore, we conclude that imputed SNPs, based upon HapMap samples, should be used for building HLA predictive models.

### Genotyping Technologies

Exploring four different genotyping technologies used in WTCCC, we have found that the concordances of genotypes between technologies are quite high in general, except for the case where genotypes are observed from one technology but are compared with imputed genotypes with data obtained from a different technology. Even in this worst case scenario, the concordances are 98.5% or higher. Further, accuracies of prediction for models built with imputed SNPs, based upon any of four technologies, are comparable with each other. Comparing the accuracies trained using genotypes of one technology and validated using another shows that the results are generally the best when the training and validation sets are the same, as expected. When they are different, Illumina 550K is slightly favored, although differences are quite modest. Overall, the prediction accuracies are comparable and the predictive models are transferable across genotyping technologies.

### Transferability of Predictive Models

The validity of constructed predictive models hinges upon the local LD, which is influenced by evolutionary history and related population genetic parameters such as recombination process and mutations, among HLA and SNP alleles [9]. Presumably, such LD would be higher within ethnic groups, and a bit lower between ethnic groups. Empirical results from our explorations suggest that predictive models for intermediate resolution HLA alleles have fairly high accuracies across different study populations of Caucasians. For high resolution HLA alleles, predictive models for HLA-A, -C and -DQB1 seem to maintain fairly decent accuracies across populations. However, for HLA-B and HLA-DRB1, predictive models seem to have much reduced accuracies in a different population, probably because genetic variations in these two loci are much more recent, negatively impacting their local LD structures. Given the limited availability of data, we were unable to evaluate the same question in different ethnic groups.

### Use of Multi-Ethnic Samples

Our exploration has supported the idea that mixing multi-ethnic samples into the training set ensures the presence of more HLA alleles and results in universal predictive models that tend to perform generally well. Of course, for situations where many samples, such as Caucasian samples, are available, ethnic-specific predictive models probably outperform universal models, although improvements are modest. In conclusion, unless a large population of the ethnic-specific training set is available, combining all available ethnic samples as the training set likely yields preferred predictive models for most ethnic groups.

While our empirical explorations have yielded suggestive insights to those questions of interest, it is important to recognize several inherent weaknesses, which may influence interpretations of our results. First, due to empirical nature, our conclusions should be largely restricted to those pertinent populations. To generalize our observations, it would be desirable to explore other population-based studies, in particular, on ethnic groups not being represented here. Second, sample sizes, especially of ethnic groups other than Caucasian, are quite small. Given greater genetic diversity in other ethnic groups, especially African, it would be desirable to expand the current explorations to those under-studied ethnic populations. Third, HLA allelic coding in these three studies is not entirely consistent, and genotype quality is also variable, due to historically different HLA genotyping technologies. While the best effort has been made to homogenize HLA alleles, it would be desirable for future efforts to repeat the model building process with more homogeneous HLA data.

Based upon empirical results and critiques, we have chosen to combine all available samples to build "state of the art" predictive models for HLA-A, -B, -C, -DRB1, -DQB1 and -DPB1. For the ease of their applications, we provide a prediction program which can take SNP data as input and generate predicted HLA alleles. Prediction accuracies are expected to be quite high for intermediate and high resolution HLA-A, -B, and -C alleles among Caucasian samples. However, it is important to note that accuracies for common HLA alleles are generally even higher (Table 7). For example, for HLA-A high resolution, when the minimum allele frequency is raised from 0 to 0.05, the prediction accuracy for WTCCC, of predictive models trained on FHCRC-CEU, improves from 97% to 99% with CT = 0.

**Table 7:** Prediction accuracies of HLA alleles with frequency exceeding a threshold

Training set		Intermediate Resolution			High Resolution		
	HLA-	0.05*	0.03	0	0.05	0.03	0
Caucasians in the FHCRC cohort (N = 1280)	A	99	99	98	99	99	97
	B	98	97	96	97	97	94
	C	97	96	96	97	97	96
	DRB1	97	97	97	91	91	88
	DQB1	99	99	99	98	98	98

Caucasians in the STEP cohort (N = 832)	A	99	99	95	99	98	95
	B	97	97	95	97	97	93
	C	96	94	94	95	95	94
	DRB1	98	98	98	98	95	91

* Threshold for HLA allele frequency

The accuracies of HLA predictive models, built with a training set of Caucasian samples from the FHCRC cohort

(genotyped on Affy 5.0) or the STEP cohort (genotyped on Illumina 1.2M), were evaluated using the WTCCC samples

(genotyped on Affy 500K) as the validation set (N = 1480). Only HLA alleles with frequency more than threshold

0.05, 0.03 or 0 in the validation set were evaluated for their prediction accuracies. The confidence threshold was set at CT = 0.

## Conclusions

Through empirical explorations, we have demonstrated that accuracies of predicting HLA alleles using SNPs are generally above 90% and some are approaching 99%, depending on HLA loci and resolution. Incorporating imputed SNPs generally improves prediction accuracies and enables universal predictive models regardless of genotyping technology. We also noticed that prediction accuracies are comparable between different genotyping technologies and the predictive models are transferable across genotyping technologies or populations from the same ethnicity. Although ethnic-specific predictive models are generally preferred, the empirical data shows that predictive models built on mixing multi-ethnic samples also perform well, providing useful predictive models when ethnic-specific models are not available.

## Availability and Requirements

Project name: Multi-allelic gene predictions using unphased SNP data.

Project home page: http://qge.fhcrc.org/MAGprediction/

Operating system(s): Linux (64 bit), Windows (64 bit)

Programming language: MATLAB 2010b, C

Other requirements: The MATLAB Compiler Runtime (MCR) 7.14, which is provided in the above website, needs to be installed on the system.

License: None.

Any restrictions to use by non-academics: No restrictions.

## Authors' contributions

XCZ participated in the initial discussion of this project, performed all data analysis, and participated in drafting the manuscript; SSL contributed to the initial conception of the project, to assisting the data analysis, and to preparation of the manuscript; HW contributed to the preparation of results for presentation; JAH. contributed to the original idea, and helped the manuscript drafting; LPZ conceived the idea, and lead the project. All authors read and approved the final manuscript.

## Supplementary Material

Additional file 1 **Supplemental data**. Detailed results of HLA alleles in the three study cohorts, prediction accuracies and the list of selected SNPs in the final HLA predictive models. Click here for file
