# Supplementary material for: Empirical evaluations of analytical issues arising from predicting HLA alleles using multiple SNPs
Source: BMC Genet. 2011 Apr 25;12:39. doi: 10.1186/1471-2156-12-39 (PMC3111398; doi:10.1186/1471-2156-12-39)
Supplement: Additional file 1 — Supplemental data. Detailed results of HLA alleles in the three study cohorts, prediction accuracies and the list of selected SNPs in the final HLA predictive models. [file 1471-2156-12-39-S1.DOCX]

**Table S1a. Distributions (%) of ambiguous alleles in three study cohorts**

@ The HLA allele frequency of the WTCCC cohort was calculated based on samples genotyped on Illumina 1.2M. Data recoding for the WTCCC cohort was from <https://www-gene.cimr.cam.ac.uk/todd/public_data/HLA/HLA.shtml>

# Standard ambiguous HLA tying combinations. # refers to other rare alleles which are not included in this list. <http://www.ebi.ac.uk/imgt/hla/pdf/ambiguity_v2280.pdf>

**Table S1b. Re-coding schema for HLA alleles in the FHCRC, WTCCC, and STEP cohort** Re-coding was to homogenize HLA alleles in the three study cohorts, based on the distributions of ambiguous alleles shown in Table S1a.

| HLA-A* | | HLA-B* | | HLA-C* | | HLA-DRB1* | | HLA-DQB1* | |
| --- | --- | --- | --- | --- | --- | --- | --- | --- | --- |
| original^@^ | new | original | new | original | new | original | new | original | new |
| 0201/0224/02G1/02 | 0292 | 0702/07G1 | 0791 | 0401/04G1 | 0491 | 0101/0102/0103/0104/01 | 0192 | 0201/0202/02AB/02 | 0292 |
| 0301/03G1 | 0391 | 1501/15G1 | 1591 | 0701/0702/07G1/07 | 0792 | 0301/0302/03 | 0392 | 06XX | 06 |
| 2402/24G1 | 2491 | 1801/18G1 | 1891 |  |  | 0801/0802/0804/08 | 0892 | Q1/Q2 | 00 |
| 3301/3303/33 | 3392 | 3501/3503/35G1/35 | 3592 |  |  | 1101/1104/11 | 1192 |  |  |
| 6801/68G1 | 6891 | 4001/40G1 | 4091 |  |  | 1301/1302/13 | 1392 |  |  |
|  |  | 4402/44G1 | 4491 |  |  | 1501/1502/15XX/15 | 1592 |  |  |
|  |  | 5101/51G1 | 5191 |  |  |  |  |  |  |

@ original allele coding in any of the three study cohorts

**Table S2. Usefulness of imputed SNPs from four different genotyping arrays for HLA predictive models** Comparison of prediction accuracies between models built with and without imputed SNPs from four different genotyping arrays across HLA-A, -B, -C, -DRB1 and -DQB1 at intermediate and high resolution, using the WTCCC control data. The training set (N=501) and validation set (N=500) were the same among the four arrays. The confidence threshold (CT) was set at 0, 0.5, and 0.9. On average, using imputed SNPs improves the prediction accuracy (call rate) by 0, 0.2%(-0.2%), 0(-2.4%) at intermediate resolution and 0.2%, 0.8(-2%), -0.4%(4%) at high resolution for Affy 500K; 0.6%, 0.6%(-0.6%), 0.4%(0) at intermediate resolution and 0, 0.2(-1.2%), -0.4%(2.2%) at high resolution for Affy 6.0; 0.6%, 0.2%(0.2%), -0.2%(-3.2%) at intermediate resolution and 0.6%, 0.8(-0.6%), 0(1%) at high resolution for Illumina 550K; 0.4%, 0.4%(-0.2%), 0(1.8%) at intermediate resolution and -0.2%, 0(-0.2%), -0.6%(0.8%) at high resolution for Illumina 1.2M.

|  | |  |  | Without Imputed SNPs | | | With Imputed SNPs | | |
| --- | --- | --- | --- | --- | --- | --- | --- | --- | --- |
|  |  | HLA- | N^#^ | CT=0 | CT=0.5 | CT=0.9 | CT=0 | CT=0.5 | CT=0.9 |
| Affy  500K | Intermediate  Resolution | A | 501 | 97 | 97(100)* | 99(90) | 97 | 97(99) | 99(83) |
|  |  | B | 501 | 95 | 95(98) | 97(90) | 95 | 96(98) | 98(82) |
|  |  | C | 501 | 95 | 95(100) | 96(90) | 96 | 96(100) | 96(94) |
|  |  | DRB1 | 496 | 97 | 98(100) | 99(95) | 97 | 97(100) | 98(97) |
|  |  | DQB1 | 498 | 99 | 99(99) | 99(97) | 98 | 99(99) | 99(94) |
|  | High Resolution | A | 500 | 97 | 97(99) | 99(88) | 96 | 96(100) | 98(86) |
|  |  | B | 493 | 94 | 94(97) | 97(84) | 94 | 95(98) | 96(87) |
|  |  | C | 492 | 94 | 95(99) | 96(75) | 93 | 93(100) | 96(88) |
|  |  | DRB1 | 488 | 90 | 91(97) | 99(50) | 91 | 95(83) | 99(52) |
|  |  | DQB1 | 470 | 96 | 96(99) | 98(91) | 98 | 98(100) | 98(95) |
| Affy  6.0 | Intermediate  Resolution | A | 501 | 97 | 97(100) | 99(88) | 98 | 98(98) | 99(90) |
|  |  | B | 501 | 94 | 95(98) | 97(87) | 96 | 96(98) | 98(87) |
|  |  | C | 501 | 96 | 96(100) | 96(93) | 96 | 96(100) | 96(95) |
|  |  | DRB1 | 496 | 98 | 98(100) | 98(97) | 98 | 98(100) | 99(97) |
|  |  | DQB1 | 498 | 98 | 98(100) | 99(97) | 98 | 99(99) | 99(93) |
|  | High Resolution | A | 500 | 97 | 97(100) | 99(87) | 97 | 97(97) | 99(86) |
|  |  | B | 493 | 94 | 94(99) | 97(81) | 94 | 95(96) | 97(84) |
|  |  | C | 492 | 94 | 94(100) | 95(78) | 95 | 95(100) | 95(86) |
|  |  | DRB1 | 488 | 94 | 94(99) | 98(56) | 93 | 93(99) | 97(56) |
|  |  | DQB1 | 470 | 97 | 97(99) | 98(95) | 97 | 97(99) | 97(96) |
| Illumina 550K | Intermediate  Resolution | A | 501 | 98 | 98(100) | 100(89) | 98 | 98(100) | 99(85) |
|  |  | B | 501 | 94 | 95(98) | 98(84) | 96 | 96(99) | 98(81) |
|  |  | C | 501 | 96 | 96(100) | 96(93) | 96 | 96(100) | 97(91) |
|  |  | DRB1 | 496 | 98 | 98(100) | 99(98) | 98 | 98(100) | 98(94) |
|  |  | DQB1 | 498 | 98 | 99(99) | 99(98) | 99 | 99(99) | 99(95) |
|  | High Resolution | A | 500 | 98 | 98(99) | 99(85) | 98 | 98(99) | 99(86) |
|  |  | B | 493 | 92 | 93(97) | 96(86) | 95 | 95(99) | 96(86) |
|  |  | C | 492 | 95 | 95(100) | 96(89) | 95 | 95(99) | 95(89) |
|  |  | DRB1 | 488 | 94 | 95(99) | 97(60) | 94 | 95(96) | 97(61) |
|  |  | DQB1 | 470 | 97 | 97(100) | 97(93) | 97 | 98(99) | 98(96) |
| Illumina 1.2M | Intermediate  Resolution | A | 501 | 97 | 97(100) | 99(90) | 99 | 99(96) | 99(91) |
|  |  | B | 501 | 97 | 97(99) | 98(89) | 97 | 97(99) | 98(90) |
|  |  | C | 501 | 97 | 97(100) | 97(90) | 97 | 97(100) | 97(92) |
|  |  | DRB1 | 496 | 99 | 99(99) | 99(94) | 99 | 99(100) | 99(97) |
|  |  | DQB1 | 498 | 99 | 99(99) | 99(97) | 99 | 99(100) | 99(99) |
|  | High Resolution | A | 500 | 97 | 97(100) | 99(89) | 97 | 97(100) | 99(89) |
|  |  | B | 493 | 95 | 95(99) | 98(86) | 95 | 95(99) | 97(87) |
|  |  | C | 492 | 95 | 95(100) | 96(91) | 95 | 95(100) | 96(89) |
|  |  | DRB1 | 488 | 93 | 93(98) | 98(58) | 94 | 95(97) | 98(61) |
|  |  | DQB1 | 470 | 99 | 99(100) | 99(97) | 97 | 97(100) | 97(95) |

# Number of samples in the validation set, samples with missing HLA data are excluded

* Prediction accuracy % (call rate %)

**Table S3. Accuracies of cross-platform predictions** Comparison of four genotyping platforms using the WTCCC control data, with respect to cross-platform predictions, i.e., training model using SNP data from one platform and validating using SNP data from another platform. Both observed and imputed HapMap SNPs were used in training and validation processes. The training set (N=501) and validation set (N=500) were the same among the four platforms. The confidence threshold (CT) was set at 0, 0.5, and 0.9.

| training/validation | |  | Affy 500K | | | Affy 6.0 | | | Illumina 550K | | | Illumina 1.2M | | |
| --- | --- | --- | --- | --- | --- | --- | --- | --- | --- | --- | --- | --- | --- | --- |
|  |  | HLA- | CT=0 | CT=0.5 | CT=0.9 | CT=0 | CT=0.5 | CT=0.9 | CT=0 | CT=0.5 | CT=0.9 | CT=0 | CT=0.5 | CT=0.9 |
| Affy 500K | Intermediate  resolution | A | 97 | 97(99) | 99(83) | 96 | 96(99) | 99(83) | 95 | 95(99) | 99(84) | 96 | 96(99) | 99(86) |
|  |  | B | 95 | 96(98) | 98(82) | 95 | 95(98) | 97(84) | 94 | 95(99) | 97(82) | 94 | 94(99) | 97(86) |
|  |  | C | 96 | 96(100) | 96(94) | 96 | 96(100) | 96(96) | 96 | 96(99) | 97(93) | 96 | 96(100) | 97(94) |
|  |  | DRB1 | 97 | 97(100) | 98(97) | 98 | 98(100) | 98(97) | 97 | 97(100) | 98(97) | 98 | 98(100) | 98(99) |
|  |  | DQB1 | 98 | 99(99) | 99(94) | 98 | 98(98) | 99(95) | 98 | 98(97) | 99(90) | 99 | 99(100) | 100(96) |
|  | High resolution | A | 96 | 96(100) | 98(86) | 95 | 95(100) | 99(89) | 94 | 94(99) | 99(84) | 95 | 96(99) | 98(85) |
|  |  | B | 94 | 95(98) | 96(87) | 94 | 95(98) | 96(89) | 94 | 94(98) | 96(87) | 94 | 94(99) | 96(88) |
|  |  | C | 93 | 93(100) | 96(88) | 93 | 93(100) | 94(90) | 93 | 93(100) | 96(85) | 94 | 94(100) | 96(87) |
|  |  | DRB1 | 91 | 95(83) | 99(52) | 92 | 94(84) | 99(52) | 92 | 94(85) | 97(53) | 93 | 95(85) | 99(54) |
|  |  | DQB1 | 98 | 98(100) | 98(95) | 96 | 97(99) | 98(93) | 97 | 97(100) | 97(93) | 97 | 97(100) | 98(95) |
| Affy 6.0 | Intermediate  resolution | A | 97 | 98(96) | 99(88) | 98 | 98(98) | 99(90) | 97 | 97(99) | 99(91) | 97 | 98(96) | 100(88) |
|  |  | B | 96 | 96(98) | 98(86) | 96 | 96(98) | 98(87) | 96 | 96(98) | 98(85) | 96 | 96(98) | 98(86) |
|  |  | C | 95 | 95(100) | 96(92) | 96 | 96(100) | 96(95) | 96 | 96(100) | 97(93) | 96 | 96(100) | 96(95) |
|  |  | DRB1 | 97 | 97(100) | 98(95) | 98 | 98(100) | 99(97) | 98 | 98(99) | 98(96) | 98 | 98(100) | 98(99) |
|  |  | DQB1 | 98 | 99(100) | 100(94) | 98 | 99(99) | 99(93) | 98 | 98(99) | 99(94) | 99 | 99(100) | 99(97) |
|  | High resolution | A | 96 | 97(96) | 99(83) | 97 | 97(97) | 99(86) | 96 | 96(99) | 99(83) | 97 | 97(100) | 99(85) |
|  |  | B | 94 | 95(96) | 97(78) | 94 | 95(96) | 97(84) | 94 | 94(98) | 97(82) | 94 | 94(98) | 97(83) |
|  |  | C | 94 | 94(100) | 96(83) | 95 | 95(100) | 95(86) | 95 | 95(100) | 96(88) | 95 | 95(100) | 96(89) |
|  |  | DRB1 | 92 | 93(98) | 98(54) | 93 | 93(99) | 97(56) | 93 | 94(98) | 97(55) | 94 | 94(98) | 97(56) |
|  |  | DQB1 | 97 | 97(100) | 97(96) | 97 | 97(99) | 97(96) | 97 | 97(100) | 97(93) | 97 | 97(100) | 97(95) |
| Illumina 550K | Intermediate  resolution | A | 97 | 98(96) | 99(85) | 98 | 98(98) | 99(74) | 98 | 98(100) | 99(85) | 99 | 99(96) | 99(86) |
|  |  | B | 95 | 95(99) | 97(77) | 96 | 96(99) | 98(80) | 96 | 96(99) | 98(81) | 96 | 96(99) | 98(80) |
|  |  | C | 96 | 96(93) | 97(77) | 96 | 96(95) | 96(81) | 96 | 96(100) | 97(91) | 96 | 96(100) | 97(93) |
|  |  | DRB1 | 97 | 97(100) | 97(97) | 98 | 98(100) | 98(96) | 98 | 98(100) | 98(94) | 98 | 98(100) | 98(98) |
|  |  | DQB1 | 99 | 99(100) | 99(97) | 99 | 99(100) | 99(97) | 99 | 99(99) | 99(95) | 99 | 99(100) | 99(99) |
|  | High resolution | A | 96 | 97(97) | 98(83) | 97 | 97(98) | 98(84) | 98 | 98(99) | 99(86) | 98 | 99(96) | 99(87) |
|  |  | B | 94 | 95(97) | 95(81) | 94 | 94(98) | 96(81) | 95 | 95(99) | 96(86) | 95 | 95(99) | 96(87) |
|  |  | C | 94 | 94(97) | 96(77) | 94 | 95(98) | 95(80) | 95 | 95(99) | 95(89) | 95 | 95(100) | 95(90) |
|  |  | DRB1 | 92 | 95(89) | 98(47) | 93 | 95(93) | 97(53) | 94 | 95(96) | 97(61) | 94 | 95(95) | 97(63) |
|  |  | DQB1 | 96 | 97(99) | 98(92) | 96 | 97(99) | 98(93) | 97 | 98(99) | 98(96) | 97 | 97(99) | 98(96) |
| Illumina 1.2M | Intermediate  resolution | A | 97 | 98(97) | 99(89) | 98 | 98(98) | 99(90) | 98 | 98(99) | 99(91) | 99 | 99(96) | 99(91) |
|  |  | B | 95 | 96(98) | 98(85) | 95 | 96(98) | 98(86) | 96 | 96(99) | 98(88) | 97 | 97(99) | 98(90) |
|  |  | C | 95 | 95(99) | 97(85) | 96 | 96(99) | 97(89) | 96 | 96(99) | 97(89) | 97 | 97(100) | 97(92) |
|  |  | DRB1 | 98 | 98(100) | 99(95) | 98 | 98(99) | 99(94) | 98 | 98(99) | 98(94) | 99 | 99(100) | 99(97) |
|  |  | DQB1 | 97 | 98(99) | 99(96) | 97 | 97(99) | 99(95) | 97 | 98(99) | 99(94) | 99 | 99(100) | 99(99) |
|  | High resolution | A | 96 | 96(98) | 99(82) | 96 | 96(100) | 98(84) | 96 | 97(99) | 99(87) | 97 | 97(100) | 99(89) |
|  |  | B | 91 | 92(97) | 96(80) | 92 | 92(97) | 96(78) | 94 | 94(100) | 97(85) | 95 | 95(99) | 97(87) |
|  |  | C | 95 | 94(99) | 96(76) | 95 | 95(100) | 95(78) | 94 | 95(100) | 96(89) | 95 | 95(100) | 96(89) |
|  |  | DRB1 | 93 | 94(94) | 98(53) | 92 | 94(95) | 98(53) | 93 | 95(94) | 97(58) | 94 | 95(97) | 98(61) |
|  |  | DQB1 | 94 | 94(99) | 96(89) | 93 | 94(99) | 96(87) | 96 | 96(99) | 97(90) | 97 | 97(100) | 97(95) |

* Prediction accuracy % (call rate %)

**Table S4. Accuracies of cross-ethnicity predictions** Comparison of accuracies of predictive models using multi-ethnicity (N=450) or Caucasian only samples (N=411) from the STEP cohort, using both observed and imputed HapMap SNPs. The confidence threshold was set at CT=0, 0.5, and 0.9. When validating on the Caucasian samples in WTCCC, on average among the HLA-A, -B, -C and -DRB1 loci, using multi-ethnic groups reduces the prediction accuracy (call rate) by 0.25%, 0.25%(0.25%), and 0.75%(1.25%) at intermediate resolution and 1.25%, 1.25%(1.75%), and 1.25%(12.25%) at high resolution for CT=0, 0.5 and 0.9 respectively. For ethnic groups that do not have the ethnic-specific training models, the multi-ethnic models improve the accuracy, but may reduce the call rate at larger CT. The average improvement on accuracy (call rate) of multi-ethnic models is 3.4%, 3.4%(3.6%), 3.6%(-2.6%) at intermediate resolution and 13%, 16.2%(-13.6%), 9.6%(-18.2%) for Mestizos in STEP, and is 2%, 5.2%(1.2%), 3.2%(-4.2%) at intermediate resolution and 8.6%, 7.6%(-3.2%), 9.6%(-12.6%) for other ethnic group in STEP.

| training/validation | |  | Caucasians in WTCCC | | | | Mestizos in STEP | | | | Other Ethnicity in STEP | | | |
| --- | --- | --- | --- | --- | --- | --- | --- | --- | --- | --- | --- | --- | --- | --- |
|  |  | HLA- | N^#^ | CT=0 | CT=0.5 | CT=0.9 | N | CT=0 | CT=0.5 | CT=0.9 | N | CT=0 | CT=0.5 | CT=0.9 |
| STEP multi-ethnicity | Intermediate resolution | A | 1712 | 97 | 97(98)* | 99(90) | 62 | 99 | 99(100) | 99(95) | 59 | 96 | 97(98) | 97(73) |
|  |  | B | 1604 | 97 | 97(99) | 98(83) | 62 | 94 | 95(95) | 100(60) | 59 | 87 | 87(100) | 93(61) |
|  |  | C | 1359 | 97 | 97(100) | 97(94) | 62 | 100 | 100(100) | 100(95) | 59 | 100 | 100(100) | 100(97) |
|  |  | DRB1 | 1750 | 96 | 96(99) | 96(94) | 62 | 98 | 98(98) | 98(90) | 59 | 90 | 93(95) | 97(76) |
|  |  | DPB1 |  |  |  |  | 62 | 97 | 97(100) | 99(79) | 59 | 92 | 92(100) | 92(78) |
|  | High resolution | A | 1709 | 95 | 96(97) | 97(66) | 62 | 80 | 82(87) | 84(26) | 59 | 86 | 86(100) | 94(59) |
|  |  | B | 1578 | 93 | 93(94) | 94(66) | 62 | 58 | 68(53) | 72(26) | 59 | 71 | 75(81) | 83(39) |
|  |  | C | 1333 | 96 | 96(100) | 97(92) | 62 | 97 | 97(100) | 97(94) | 59 | 97 | 96(97) | 98(92) |
|  |  | DRB1 | 1719 | 91 | 92(92) | 96(63) | 62 | 82 | 87(79) | 86(35) | 59 | 85 | 90(83) | 94(56) |
|  |  | DPB1 |  |  |  |  | 62 | 97 | 97(100) | 98(81) | 59 | 90 | 90(95) | 91(66) |
| STEP Caucasians | Intermediate resolution | A | 1712 | 97 | 97(98) | 99(90) | 62 | 98 | 98(100) | 98(95) | 59 | 93 | 93(100) | 94(97) |
|  |  | B | 1604 | 96 | 96(100) | 98(91) | 62 | 83 | 82(82) | 87(68) | 59 | 80 | 79(97) | 83(73) |
|  |  | C | 1359 | 96 | 96(97) | 97(89) | 62 | 100 | 100(98) | 100(95) | 59 | 99 | 99(100) | 100(95) |
|  |  | DRB1 | 1750 | 99 | 99(100) | 99(96) | 62 | 96 | 98(97) | 98(92) | 59 | 90 | 90(93) | 95(78) |
|  |  | DPB1 |  |  |  |  | 62 | 94 | 94(98) | 95(82) | 59 | 81 | 82(97) | 91(63) |
|  | High resolution | A | 1709 | 96 | 96(98) | 98(88) | 62 | 69 | 69(100) | 67(87) | 59 | 81 | 83(97) | 84(85) |
|  |  | B | 1578 | 95 | 96(99) | 97(88) | 62 | 38 | 38(89) | 50(48) | 59 | 71 | 72(93) | 78(69) |
|  |  | C | 1333 | 96 | 96(100) | 97(92) | 62 | 97 | 97(100) | 98(97) | 59 | 93 | 93(100) | 93(97) |
|  |  | DRB1 | 1719 | 93 | 94(93) | 97(68) | 62 | 68 | 68(100) | 88(42) | 59 | 61 | 66(85) | 72(51) |
|  |  | DPB1 |  |  |  |  | 62 | 77 | 78(98) | 76(79) | 59 | 80 | 79(97) | 85(73) |

# Number of samples in the validation dataset

* Prediction accuracy % (call rate %)

**Table S5. List of the selected SNPs in the final HLA predictive models**

Intermediate Resolution

| A | | B | | C | | DRB1 | | DQB1 | | DPB1 | |
| --- | --- | --- | --- | --- | --- | --- | --- | --- | --- | --- | --- |
| rs | pos | rs | pos | rs | pos | rs | pos | rs | pos | rs | pos |
| rs12110682 | 29850894 | rs1265180 | 31267324 | rs2894180 | 31280634 | rs12180571 | 32506503 | rs3129933 | 32444139 | rs3130171 | 33106707 |
| rs7763677 | 29894485 | rs1265178 | 31269208 | rs3095250 | 31316319 | rs17496549 | 32517686 | rs2894254 | 32453667 | rs7774158 | 33115730 |
| rs9258554 | 29920538 | rs3906273 | 31370902 | rs3130688 | 31318195 | rs3763326 | 32521535 | rs7451962 | 32690413 | rs9277341 | 33147603 |
| rs11759549 | 29925902 | rs11760024 | 31371685 | rs6901869 | 31318244 | rs16822660 | 32521538 | rs17533167 | 32698822 | rs2071353 | 33152235 |
| rs2734985 | 29926641 | rs12182794 | 31377863 | rs1639106 | 31321296 | rs13209234 | 32523953 | rs2187668 | 32713862 | rs2071354 | 33152366 |
| rs9258605 | 29928089 | rs7761965 | 31381474 | rs3130409 | 31321790 | rs9268831 | 32535726 | rs9272535 | 32714734 | rs3135021 | 33153536 |
| rs6919513 | 29931973 | rs2508004 | 31381574 | rs3095241 | 31323614 | rs9269182 | 32555835 | rs9272723 | 32717405 | rs3135024 | 33155444 |
| rs9258690 | 29932319 | rs1634761 | 31382006 | rs9501543 | 31324935 | rs9269190 | 32556478 | rs6928482 | 32734227 | rs928976 | 33157189 |
| rs9258876 | 29945051 | rs3094691 | 31382672 | rs2894189 | 31325794 | rs9391786 | 32556539 | rs3891175 | 32742445 | rs2856827 | 33157252 |
| rs9258881 | 29945121 | rs981536 | 31390233 | rs1639114 | 31327203 | rs7749057 | 32556882 | rs3134975 | 32760559 | rs9378176 | 33157287 |
| rs3893468 | 29945861 | rs2844586 | 31426003 | rs2524121 | 31336951 | rs2157339 | 32619650 | rs2856683 | 32763196 | rs9378177 | 33157362 |
| rs2844821 | 29946621 | rs2523619 | 31426123 | rs2248902 | 31342093 | rs9270986 | 32682038 | rs7774573 | 32765605 | rs9500928 | 33157672 |
| rs2523809 | 29957598 | rs4394275 | 31426156 | rs9264608 | 31345639 | rs615672 | 32682149 | rs9405119 | 32765659 | rs7772134 | 33157704 |
| rs2523807 | 29958253 | rs2523618 | 31426282 | rs7767581 | 31347834 | rs660895 | 32685358 | rs7775228 | 32766057 | rs9277366 | 33158085 |
| rs9468650 | 29975618 | rs1058026 | 31429664 | rs6457358 | 31347978 | rs6921236 | 32685910 | rs2858330 | 32766693 | rs9277377 | 33158181 |
| rs9259775 | 29997873 | rs2523608 | 31430538 | rs2844622 | 31348039 | rs522308 | 32689900 | rs2647015 | 32772071 | rs9277379 | 33158303 |
| rs2524005 | 30007656 | rs3819284 | 31430746 | rs2074489 | 31348107 | rs7451962 | 32690413 | rs9275332 | 32774921 | rs9277381 | 33158372 |
| rs2860580 | 30014670 | rs2523589 | 31435313 | rs5009853 | 31348615 | rs9271366 | 32694832 | rs9275390 | 32777134 | rs3128960 | 33158632 |
| rs7745413 | 30023448 | rs2523586 | 31435414 | rs6923313 | 31349349 | rs3129763 | 32698903 | rs11752643 | 32777351 | rs3097674 | 33158661 |
| rs417162 | 30024484 | rs1811197 | 31435639 | rs2524084 | 31349618 | rs4639334 | 32710192 | rs9275418 | 32778222 | rs9277394 | 33158948 |
| rs1655903 | 30024984 | rs2596477 | 31435702 | rs2844615 | 31350938 | rs9272219 | 32710247 |  |  | rs9277395 | 33159029 |
| rs2571400 | 30035701 | rs2523567 | 31437994 | rs13203895 | 31352061 | rs2040410 | 32710676 |  |  | rs9277396 | 33159117 |
| rs2517679 | 30041148 | rs2596548 | 31438525 | rs10456057 | 31353513 | rs6928482 | 32734227 |  |  | rs9277409 | 33159602 |
| rs2523971 | 30046237 | rs2523560 | 31438823 | rs6906846 | 31353715 | rs3134975 | 32760559 |  |  | rs9277410 | 33159618 |
| rs1264813 | 30047879 | rs9266340 | 31439285 | rs7381988 | 31354682 | rs7774573 | 32765605 |  |  | rs9277412 | 33159667 |
| rs2844805 | 30048658 | rs2523554 | 31439808 | rs9368669 | 31356472 | rs9275293 | 32771286 |  |  | rs9277421 | 33159798 |
| rs7770569 | 30049518 | rs9266355 | 31440127 | rs2524055 | 31360216 | rs9275329 | 32774809 |  |  | rs9277424 | 33159843 |
| rs2571375 | 30053249 | rs7766461 | 31442778 | rs2853930 | 31363403 | rs2647045 | 32776078 |  |  | rs9277426 | 33159888 |
| rs2246199 | 30053599 | rs2596429 | 31442800 | rs2524048 | 31364540 | rs9275390 | 32777134 |  |  | rs9277431 | 33160006 |
| rs11759891 | 30060126 | rs6457401 | 31442831 | rs16899160 | 31364646 | rs9275418 | 32778222 |  |  | rs9277432 | 33160050 |
| rs9260989 | 30070496 | rs2922994 | 31443880 | rs9468925 | 31366816 | rs9275427 | 32778893 |  |  | rs9277434 | 33160164 |
| rs3115631 | 30094303 | rs7743761 | 31444079 | rs2524162 | 31367729 | rs9391744 | 32788205 |  |  | rs9277437 | 33160228 |
| rs9261200 | 30111284 | rs9266404 | 31444202 | rs2844579 | 31441695 | rs3104395 | 32793404 |  |  | rs9277450 | 33160673 |
|  |  | rs2523535 | 31444229 | rs9266688 | 31456522 |  |  |  |  | rs9277458 | 33161145 |
|  |  | rs9266406 | 31444397 | rs9266701 | 31457009 |  |  |  |  | rs9277463 | 33161285 |
|  |  | rs12660817 | 31444736 | rs4293988 | 31458359 |  |  |  |  | rs9277464 | 33161330 |
|  |  | rs2844559 | 31448054 |  |  |  |  |  |  | rs9277468 | 33161433 |
|  |  | rs9266513 | 31449275 |  |  |  |  |  |  | rs9277469 | 33161446 |
|  |  | rs4463302 | 31451646 |  |  |  |  |  |  | rs9277470 | 33161455 |
|  |  | rs4959068 | 31451823 |  |  |  |  |  |  | rs9277479 | 33161767 |
|  |  | rs3997982 | 31452273 |  |  |  |  |  |  | rs9277489 | 33161920 |
|  |  | rs9266636 | 31455012 |  |  |  |  |  |  | rs9277492 | 33161960 |
|  |  | rs3094600 | 31455123 |  |  |  |  |  |  | rs9277497 | 33162069 |
|  |  | rs4713462 | 31455795 |  |  |  |  |  |  | rs9277515 | 33162246 |
|  |  | rs9266669 | 31456056 |  |  |  |  |  |  | rs9277518 | 33162280 |
|  |  | rs2596568 | 31458211 |  |  |  |  |  |  | rs1042448 | 33162320 |
|  |  | rs2507976 | 31459866 |  |  |  |  |  |  | GA023808 | 33162411 |
|  |  | rs9266774 | 31460859 |  |  |  |  |  |  | rs1042544 | 33162435 |
|  |  | rs4081552 | 31461668 |  |  |  |  |  |  | rs929 | 33162597 |
|  |  | rs2523483 | 31461771 |  |  |  |  |  |  | rs9277533 | 33162699 |
|  |  | rs9266777 | 31462179 |  |  |  |  |  |  | rs9277534 | 33162785 |
|  |  | rs4349859 | 31473766 |  |  |  |  |  |  | rs9277535 | 33162839 |
|  |  | rs2523454 | 31475844 |  |  |  |  |  |  | rs9277538 | 33163025 |
|  |  | rs9266816 | 31489512 |  |  |  |  |  |  | rs9501255 | 33163076 |
|  |  | rs9469002 | 31515751 |  |  |  |  |  |  | rs9277540 | 33163101 |
|  |  | rs9469003 | 31515807 |  |  |  |  |  |  | rs9501259 | 33163529 |
|  |  | rs11758964 | 31554345 |  |  |  |  |  |  | rs9296076 | 33164818 |
|  |  |  |  |  |  |  |  |  |  | rs2281390 | 33167647 |
|  |  |  |  |  |  |  |  |  |  | rs7763822 | 33168406 |
|  |  |  |  |  |  |  |  |  |  | rs2295119 | 33168848 |
|  |  |  |  |  |  |  |  |  |  | rs4412248 | 33180393 |
|  |  |  |  |  |  |  |  |  |  | rs16868789 | 33180576 |
|  |  |  |  |  |  |  |  |  |  | rs9277629 | 33190087 |
|  |  |  |  |  |  |  |  |  |  | rs2855430 | 33249258 |

High Resolution

| A | | B | | C | | DRB1 | | DQB1 | | DPB1 | |
| --- | --- | --- | --- | --- | --- | --- | --- | --- | --- | --- | --- |
| rs | pos | rs | pos | rs | pos | rs | pos | rs | pos | rs | pos |
| rs2734995 | 29918924 | rs3906273 | 31370902 | rs2233980 | 31187623 | rs3763305 | 32477466 | rs3129881 | 32517462 | rs11758154 | 33093572 |
| rs9258554 | 29920538 | rs9380237 | 31372371 | rs1265156 | 31250276 | rs3806155 | 32481356 | rs16822660 | 32521538 | rs11755748 | 33093782 |
| rs3873260 | 29921661 | rs16899213 | 31376121 | rs4122189 | 31275906 | rs9268557 | 32497283 | rs12529093 | 32523836 | rs9277053 | 33114200 |
| rs9258557 | 29921921 | rs7761965 | 31381474 | rs6904246 | 31276179 | rs3135363 | 32497626 | rs9268832 | 32535767 | rs7738815 | 33128365 |
| rs2394186 | 29924400 | rs16899241 | 31381682 | rs7768431 | 31310644 | rs2395175 | 32513004 | rs12191360 | 32559339 | rs9277341 | 33147603 |
| rs2523765 | 29925085 | rs3873386 | 31381724 | rs3130531 | 31314595 | rs3129882 | 32517508 | rs2516049 | 32678378 | rs987870 | 33150858 |
| rs3094171 | 29936769 | rs1634761 | 31382006 | rs3134768 | 31314823 | rs6911777 | 32517974 | rs615672 | 32682149 | rs2071351 | 33151908 |
| rs765649 | 29939037 | rs10484554 | 31382534 | rs3130688 | 31318195 | rs9405112 | 32553578 | rs522308 | 32689900 | rs2071352 | 33152166 |
| rs3094165 | 29941520 | rs9264947 | 31382626 | rs6901869 | 31318244 | rs9269182 | 32555835 | rs7451962 | 32690413 | rs2071353 | 33152235 |
| rs2734973 | 29941927 | rs3094691 | 31382672 | rs3130695 | 31319029 | rs2157337 | 32609122 | rs9271775 | 32702306 | rs2071354 | 33152366 |
| rs3132718 | 29944556 | rs9391764 | 31390117 | rs1639106 | 31321296 | rs2157339 | 32619650 | rs9272723 | 32717405 | rs3135021 | 33153536 |
| rs3893468 | 29945861 | rs4540292 | 31425161 | rs4332019 | 31321392 | rs9270657 | 32673999 | rs7744001 | 32734064 | rs3135024 | 33155444 |
| rs886398 | 29947247 | rs2844586 | 31426003 | rs3130409 | 31321790 | rs615672 | 32682149 | rs3891175 | 32742445 | rs928976 | 33157189 |
| rs2523809 | 29957598 | rs2523619 | 31426123 | rs2894196 | 31338090 | rs660895 | 32685358 | rs3134975 | 32760559 | rs9378176 | 33157287 |
| rs9259633 | 29983656 | rs2523618 | 31426282 | rs9264482 | 31340391 | rs6921236 | 32685910 | rs4947342 | 32761048 | rs9500928 | 33157672 |
| rs9259832 | 30003318 | rs1058026 | 31429664 | rs9264526 | 31341967 | rs7451962 | 32690413 | rs7775228 | 32766057 | rs7772134 | 33157704 |
| rs2524005 | 30007656 | rs3819294 | 31430466 | rs9264532 | 31342360 | rs9271366 | 32694832 | rs2858330 | 32766693 | rs9277366 | 33158085 |
| rs7745413 | 30023448 | rs2523608 | 31430538 | rs7767581 | 31347834 | rs17533167 | 32698822 | rs2647015 | 32772071 | rs9277377 | 33158181 |
| rs2735096 | 30023730 | rs2523589 | 31435313 | rs2844622 | 31348039 | rs3129763 | 32698903 | rs9275379 | 32776782 | rs9277379 | 33158303 |
| rs417162 | 30024484 | rs2523586 | 31435414 | rs2074488 | 31348410 | rs4639334 | 32710192 | rs11752643 | 32777351 | rs9277381 | 33158372 |
| rs1655903 | 30024984 | rs1811197 | 31435639 | rs9357121 | 31348458 | rs3891175 | 32742445 | rs2647050 | 32777745 | rs3128960 | 33158632 |
| rs11966184 | 30025919 | rs9378249 | 31435680 | rs5009853 | 31348615 | rs3134996 | 32744844 | rs9275418 | 32778222 | rs3097674 | 33158661 |
| rs9260587 | 30030471 | rs2596477 | 31435702 | rs5010528 | 31349011 | rs2858330 | 32766693 | rs2856718 | 32778233 | rs9277394 | 33158948 |
| rs9260606 | 30030796 | rs9266340 | 31439285 | rs13191343 | 31349088 | rs9275312 | 32773706 | rs9275517 | 32782627 | rs9277395 | 33159029 |
| rs9260607 | 30030835 | rs9266355 | 31440127 | rs2524084 | 31349618 | rs2856726 | 32774699 | rs9275524 | 32783087 | rs9277396 | 33159117 |
| rs9260676 | 30034118 | rs4327724 | 31440667 | rs3132491 | 31350251 | rs9275329 | 32774809 | rs17427887 | 32787085 | rs9277409 | 33159602 |
| rs9260682 | 30034455 | rs9501571 | 31441589 | rs2844615 | 31350938 | rs2858312 | 32775208 | rs12527228 | 32787970 | rs9277410 | 33159618 |
| rs7739434 | 30038598 | rs9501572 | 31441799 | rs2844611 | 31351958 | rs2647040 | 32775258 | rs2067578 | 32870439 | rs9277412 | 33159667 |
| rs2523933 | 30040271 | rs2844576 | 31442580 | rs2524070 | 31352499 | rs2647044 | 32775888 |  |  | rs9277421 | 33159798 |
| rs6457109 | 30041240 | rs7766461 | 31442778 | rs6919908 | 31352939 | rs2647045 | 32776078 |  |  | rs9277424 | 33159843 |
| rs2844806 | 30041418 | rs2596429 | 31442800 | rs6906846 | 31353715 | rs9275382 | 32776809 |  |  | rs9277426 | 33159888 |
| rs2517673 | 30045221 | rs6457401 | 31442831 | rs2524067 | 31353800 | rs9275383 | 32776824 |  |  | rs9277431 | 33160006 |
| rs2517672 | 30045241 | rs7767216 | 31443075 | rs7381988 | 31354682 | rs9275384 | 32776876 |  |  | rs9277432 | 33160050 |
| rs2523971 | 30046237 | rs9266392 | 31443382 | rs7382297 | 31355046 | rs9275438 | 32779486 |  |  | rs9277434 | 33160164 |
| rs2571377 | 30046550 | rs2523538 | 31443602 | rs6457372 | 31355100 | rs9275495 | 32781552 |  |  | rs9277437 | 33160228 |
| rs5009448 | 30048467 | rs2844571 | 31443626 | rs3873374 | 31359290 | rs7765379 | 32788906 |  |  | rs9277450 | 33160673 |
| rs2571375 | 30053249 | rs2922994 | 31443880 | rs16899160 | 31364646 | rs9275602 | 32790790 |  |  | rs9277458 | 33161145 |
| rs5025708 | 30063178 | rs2156874 | 31443955 | rs9468925 | 31366816 | rs6935940 | 32791669 |  |  | rs9277463 | 33161285 |
| rs9260952 | 30067725 | rs2523536 | 31443979 | rs3906269 | 31366947 | rs6936863 | 32792007 |  |  | rs9277464 | 33161330 |
| rs9260957 | 30068062 | rs2523535 | 31444229 | rs3906276 | 31369063 | rs9275614 | 32792235 |  |  | rs9277468 | 33161433 |
| rs9260971 | 30069492 | rs9266409 | 31444547 | rs3906273 | 31370902 | rs2858881 | 32811823 |  |  | rs9277469 | 33161446 |
| rs9260976 | 30069749 | rs9295986 | 31446507 | rs3906272 | 31370903 |  |  |  |  | rs9277470 | 33161455 |
| rs9260984 | 30070247 | rs2844559 | 31448054 | rs2894207 | 31371730 |  |  |  |  | rs9277479 | 33161767 |
| rs9261014 | 30072978 | rs6936035 | 31449135 | rs13191519 | 31373731 |  |  |  |  | rs9277489 | 33161920 |
| rs9261035 | 30074154 | rs4463302 | 31451646 | rs2524085 | 31374543 |  |  |  |  | rs9277492 | 33161960 |
| rs9261043 | 30074705 | rs4959068 | 31451823 | rs9264848 | 31379174 |  |  |  |  | rs9277497 | 33162069 |
| rs9261105 | 30082479 | rs2844549 | 31451853 | rs1634795 | 31381993 |  |  |  |  | rs9277515 | 33162246 |
| rs6940082 | 30083074 | rs2844546 | 31452636 | rs9264951 | 31383079 |  |  |  |  | rs9277518 | 33162280 |
| rs9261151 | 30095717 | rs2442736 | 31454600 | rs2596548 | 31438525 |  |  |  |  | rs1042448 | 33162320 |
| rs165255 | 30097674 | rs3094600 | 31455123 | rs12660817 | 31444736 |  |  |  |  | rs1042544 | 33162435 |
| rs9261195 | 30110400 | rs4713460 | 31455777 |  |  |  |  |  |  | rs929 | 33162597 |
| rs9261200 | 30111284 | rs9266688 | 31456522 |  |  |  |  |  |  | rs9277533 | 33162699 |
| rs9261260 | 30131331 | rs9266718 | 31457767 |  |  |  |  |  |  | rs9277534 | 33162785 |
| rs3132129 | 30135839 | rs2596568 | 31458211 |  |  |  |  |  |  | rs9277535 | 33162839 |
| rs9261266 | 30136531 | rs9266749 | 31459292 |  |  |  |  |  |  | rs9277538 | 33163025 |
| rs9261275 | 30138907 | rs2507976 | 31459866 |  |  |  |  |  |  | rs9501255 | 33163076 |
| rs3807031 | 30141863 | rs4081552 | 31461668 |  |  |  |  |  |  | rs9277540 | 33163101 |
| rs9261282 | 30143436 | rs2523483 | 31461771 |  |  |  |  |  |  | rs9501259 | 33163529 |
| rs6457144 | 30171347 | rs9266777 | 31462179 |  |  |  |  |  |  | rs3117229 | 33164047 |
|  |  | rs2428484 | 31463792 |  |  |  |  |  |  | rs9461832 | 33164362 |
|  |  | rs2523457 | 31473686 |  |  |  |  |  |  | rs9296076 | 33164818 |
|  |  | rs4349859 | 31473766 |  |  |  |  |  |  | rs2281390 | 33167647 |
|  |  | rs2844523 | 31476567 |  |  |  |  |  |  | rs2295119 | 33168848 |
|  |  | rs3763288 | 31478346 |  |  |  |  |  |  | rs16868789 | 33180576 |
|  |  | rs12660741 | 31484968 |  |  |  |  |  |  | rs9277723 | 33199432 |
|  |  | rs1063632 | 31486489 |  |  |  |  |  |  | rs3116956 | 33237049 |
|  |  | rs1051792 | 31486956 |  |  |  |  |  |  | rs2855430 | 33249258 |
|  |  | rs3819268 | 31487038 |  |  |  |  |  |  | GA005686 | 33277873 |
|  |  | rs1051794 | 31487088 |  |  |  |  |  |  | rs1704996 | 33290873 |
|  |  | rs2256026 | 31487120 |  |  |  |  |  |  |  |  |
|  |  | rs3828875 | 31487147 |  |  |  |  |  |  |  |  |
|  |  | rs6933779 | 31488711 |  |  |  |  |  |  |  |  |
|  |  | rs6932236 | 31491958 |  |  |  |  |  |  |  |  |
|  |  | rs9501106 | 31496088 |  |  |  |  |  |  |  |  |
|  |  | rs9469003 | 31515807 |  |  |  |  |  |  |  |  |
|  |  | rs9765960 | 31520979 |  |  |  |  |  |  |  |  |
|  |  | rs2395029 | 31539759 |  |  |  |  |  |  |  |  |
|  |  | rs4713466 | 31543848 |  |  |  |  |  |  |  |  |
|  |  | rs2518027 | 31544455 |  |  |  |  |  |  |  |  |
|  |  | rs2844507 | 31544560 |  |  |  |  |  |  |  |  |
|  |  | rs2395031 | 31545284 |  |  |  |  |  |  |  |  |
|  |  | rs2244839 | 31546347 |  |  |  |  |  |  |  |  |
|  |  | rs3828887 | 31548578 |  |  |  |  |  |  |  |  |
|  |  | rs2905747 | 31559455 |  |  |  |  |  |  |  |  |
|  |  | rs2428501 | 31561526 |  |  |  |  |  |  |  |  |
